# Lactose Malabsorption Testing in Daily Clinical Practice: A Critical Retrospective Analysis and Comparison of the Hydrogen/Methane Breath Test and Genetic Test (C/T_−13910_ Polymorphism) Results

**DOI:** 10.1155/2014/464382

**Published:** 2014-04-16

**Authors:** Dietmar Enko, Erwin Rezanka, Robert Stolba, Gabriele Halwachs-Baumann

**Affiliations:** Department of Laboratory Medicine, Central Hospital Steyr, Sierningerstraße 170, 4400 Steyr, Austria

## Abstract

The aim of this study was to establish a retrospective evaluation and comparison of the hydrogen/methane (H_2_/CH_4_) breath test and genetic test (C/T_−13910_ polymorphism) results in lactose malabsorption testing. In total 263 consecutive patients with suspected lactose malabsorption were included in this study. They underwent the H_2_/CH_4_ breath test following the ingestion of 50 g lactose and were tested for the C/T_−13910_ polymorphism. In total 51 patients (19.4%) had a C/C_−13910_ genotype, indicating primary lactose malabsorption. Only 19 patients (7.2%) also had a positive H_2_/CH_4_ breath test. All in all 136 patients (51.69%) had a C/T_−13910_ and 76 patients (28.91%) a T/T_−13910_ genotype, indicating lactase persistence. Four patients (1.5%) with the C/T_−13910_ genotype and one patient (0.4%) with the T/T_−13910_ genotype had a positive H_2_/CH_4_ breath test result, indicating secondary lactose malabsorption. Cohen's Kappa measuring agreement between the two methods was 0.44. Twenty patients (7.6%) with a positive H_2_/CH_4_ peak within 60 minutes after lactose ingestion were classified as patients with lactose-dependent small intestinal bacterial overgrowth (SIBO). In conclusion, only moderate agreement between the breath test and the genetic test was shown. Secondary lactose malabsorption as well as preanalytical limitations of the combined H_2_/CH_4_ breath test procedure can cause discrepant results. This trial is registered with K-42-13.

## 1. Introduction

The disaccharide lactose is synthesized in the mammary gland of mammalians (except the sea lion) and is essential for the nourishment of newborn infants [1, 2]. In the small intestine brush border the lactase enzyme is responsible for the absorption of lactose [3]. After the ingestion of lactose, the unabsorbable disaccharide is hydrolysed into the monosaccharides glucose and galactose, that are absorbed [4]. If the lactase enzyme activity is inadequate, the unabsorbed lactose will reach the large intestine, where the gut flora ferments the sugar molecules into short-chain fatty acids, carbon dioxide (CO_2_), hydrogen (H_2_), and methane (CH_4_) [5, 6]. Lactose malabsorption (hypolactasia) exists in three different forms: congenital, primary, and secondary [3, 7]. The congenital lactase deficiency is an extremely rare autosomal recessive lifelong gastrointestinal disorder, leading to watery diarrhea from the first exposure to breast milk in infants [2, 3, 8]. The primary lactose malabsorption, also known as adult-type hypolactasia or lactase nonpersistence, is the most common phenotype found in humans. This form is inherited autosomal recessive and results in a decline of lactase enzyme activity in the small intestine. A single nucleotide polymorphism (C/T_−13910_) 14 kb upstream from the lactase gene (LCT) locus is associated with the adult-type hypolactasia [2, 3, 9]. The analysis of the LCT C/T_−13910_ polymorphism is considered to have a strong concordance with the lactose breath test for predicting lactose malabsorption in European populations [10–12]. The secondary lactose malabsorption is an acquired and reversible form associated with inflammatory bowel disease, celiac and tropical sprue, short bowel syndrome, radiation enteritis, infectious enteritis, gastrointestinal surgery, drugs, or small bowel bacterial overgrowth (SIBO). Lactose malabsorption is defined as inefficient digestion due to intestinal pathologies or lactase nonpersistence, whereas lactose intolerance is defined as lactose malabsorption with gastrointestinal symptoms [5, 7]. The lactose breath test is a widely used diagnostic tool for lactose malabsorption testing. The reliability of this test depends on the activity of the intestinal bacterial flora fermenting undigested lactose and producing CO_2_, H_2,_ and CH_4_. These gases are not only absorbed and eliminated via the lungs but also can cause abdominal pain, bloating, flatulence, and diarrhea [1, 13]. Only about one-third of the lactose malabsorbers are recorded with symptoms during the breath test [14]. Despite the simple performance of the lactose breath test, the uniformity and standardization of the testing procedure and the test interpretation are still lacking [6, 15]. SIBO is a heterogeneous syndrome defined as the presence of an increased number and/or abnormal type of bacteria in the small bowel. The true prevalence of SIBO is unknown and depends on the characteristics of the diagnostic method and the study population [16, 17]. The aim of this study was to analyze the combined H_2_/CH_4_ lactose breath test and genetic test (C/T_−13910_ polymorphism) results in daily clinical practice and to compare both methods. In addition the diagnosis of lactose-dependent SIBO [6, 18] was evaluated based on the combined H_2_/CH_4_ breath test results.

## 2. Materials and Methods

### 2.1. Ethical Approval

The ethical approval for this study was provided by the Ethical Committee of Upper Austria, Linz, Austria (Trial registration number: K-42-13).

### 2.2. Patients

This retrospective study was performed at the Department of Laboratory Medicine in the Central Hospital Steyr. A total of 298 consecutive case histories of patients were reviewed. They came to the outpatient clinic from July 1, 2007, to July 31, 2010, to undergo an examination of lactose malabsorption. Inclusion and exclusion criteria were as follows. The inclusion criteria were the parallel performance of the combined H_2_/CH_4_ lactose breath test and the genetic test (C/T_−13910_ polymorphism) in the above-mentioned period, a twelve-hour overnight fasting, and abstaining from smoking. Patients who have completed antibiotic therapy were excluded for at least four weeks from the breath test. In total 35 patients were excluded from this study because they did not fulfill all the defined diagnostic criteria of the combined H_2_/CH_4_ breath test as listed below.

### 2.3. H_2_/CH_4_ Breath Test

Gas chromatography was employed to measure the breath H_2_ and CH_4_ concentration using the QuinTron Model DP Plus MicroLyzer (QuinTron, Milwaukee, WI, USA). After determining the baseline breath H_2_ and CH_4_ concentration, lactose was given in a dose of 50 g dissolved in 200 mL of water [10, 19]. The end-expiratory breath H_2_ and CH_4_ concentration was measured at 15, 30, 45, 60, 75, 90, and 120 minutes after lactose ingestion. The results were expressed in parts per million (ppm). During the test the patients were asked to report clinical symptoms and to avoid eating, smoking, and physical effort. The breath test result was considered positive if the H_2_ and/or the CH_4_ peak was >20 ppm over the baseline value [20]. Patients were classified as lactose malabsorbers if a H_2_ and/or CH_4_ increase >20 ppm over the baseline value was observed within the colon passage 60–120 minutes after the lactose ingestion [6, 21]. If lactose malabsorption was accompanied by gastrointestinal symptoms, patients were classified as lactose intolerant. Lactose-dependent SIBO was considered if an increase of >20 H_2_ and/or CH_4_ above the baseline was observed up to 60 minutes after lactose ingestion [6]. A non-H_2_ producer status was defined as a H_2_ production <5 ppm.

### 2.4. Genetic Test

In 2005 a genetic test method was established at the Department of Laboratory Medicine in the Central Hospital Steyr. Ethylenediaminetetraacetic acid (EDTA) blood samples were drawn from patients and stored at −20°C for later deoxyribonucleic acid (DNA) preparation. The DNA was purified from EDTA blood (200 *μ*L) on the MagNA Pure Compact Instrument (Roche Diagnostics, Rotkreuz, Switzerland) using the MagNA Pure Compact Nucleic Acid Isolation Kit I (Roche Diagnostics, Rotkreuz, Switzerland) according to the manufacturer's instructions. Real-time PCR with specific fluorescent labeled hybridization probes, followed by melting curve analysis for detecting the LCT C/T_−13910_ polymorphism, was performed on the LightCycler 1.1 Instrument (3-channel carousel based system; software version 3.5.3; Roche Diagnostics, Rotkreuz, Switzerland) [10, 21].

### 2.5. Statistics

Agreement between the combined H_2_/CH_4_ breath test and the genetic test was calculated using Cohen's Kappa. According to the literature [10], the lactose breath test was considered as the gold standard method. Sensitivity, specificity, and positive and negative predictive values of the genetic test (C/T_−13910_ polymorphism) were calculated compared to the H_2_/CH_4_ breath test.

## 3. Results

### 3.1. Patient Demographic Characteristics

In total 263 patients were included in this study. Of them 180 (68.4%) were female and 83 (31.6%) were male. The mean age was 42.5 ± 19.18.

### 3.2. Lactose H_2_/CH_4_ Breath Test (Table 1)

Of all the included patients (*n* = 263), 24 patients (9.1%) showed an increase of >20 H_2_ and/or CH_4_ above the baseline value within the colon passage (60–120 minutes). All in all 21 patients (8%) had a positive H_2_ breath test, 2 patients (0.7%) had a positive CH_4_ breath test, and 1 patient (0.4%) had a combined positive H_2_/CH_4_ breath test. In total 16 patients (6.1%) with reported gastrointestinal symptoms were classified as lactose intolerant; eight patients (3%) with a lack of clinical symptoms were categorized as lactose malabsorbers. No patient had an increase >20 H_2_ and/or CH_4 _ppm above the baseline value anytime during recording.

### 3.3. Comparison between the H_2_/CH_4_ Breath Test and the Genetic Test (Table 2)

All in all 51 patients (19.4%) were C/C_−13910_ homozygotes, the responsible genotype for primary lactose malabsorption. In total 136 patients (51.69%) were C/T_−13910_ heterozygotes, and 76 patients (28.91%) were T/T_−13910_ homozygotes, indicating lactase persistence. Cohen's Kappa for agreement between the H_2_/CH_4_ breath test and the genetic test was 0.44. Considering the lactose breath test as the gold standard method [10], the sensitivity of the genetic test compared to the breath test was 79%, the specificity was 87%, the positive predictive value was 60%, and the negative predictive value was 98%. All in all 32 patients (12.2%) with the C/C_−13910_ genotype showed a negative H_2_/CH_4_ breath test result. Four patients (1.5%) with the C/T_−13910_ genotype and one patient (0.4%) with the T/T_−13910_ genotype had a positive H_2_/CH_4_ breath test result, indicating a secondary lactose malabsorption form. Of them three patients had SIBO, one patient had gastroenteritis, and one patient had colitis ulcerosa. In one patient no associated gastrointestinal disease could be found in the anamnesis.

### 3.4. CH_4_ Producers and Non-H_2_ Producers

In 6 patients (2.3%), a CH_4_ >20 ppm was measured during the H_2_/CH_4_ breath test; another 16 patients (6.1%) showed a CH_4_ measurement >10–20 ppm. In 95 patients (36.1%), the H_2_ measurement was <5 ppm during the breath test. They were classified as non-H_2_ producers. Of these only one patient showed a CH_4_ measurement >20 ppm; another four patients showed a CH_4_ measurement >10–20 ppm.

### 3.5. Symptoms during the Lactose H_2_/CH_4_ Breath Test (Table 3)

During the lactose H_2_/CH_4_ breath test, 57 patients (21.7%) reported one or more symptoms. Of these abdominal pain was present in 26 patients (45.6%), bloating in 13 patients (22.8%), and diarrhea in 11 patients (19.2%). All in all 15 patients (22.8%) reported nausea, heart burn, malaise, and extraintestinal symptoms like headache or dizziness. As shown in Table 3, 16 patients (6.1%) with a positive H_2_/CH_4_ breath test reported one or more symptoms during the test: six patients reported abdominal pain, five patients bloating, four patients diarrhea, and five patients nausea, malaise, or headache. Furthermore 41 patients (15.6%) with a negative H_2_/CH_4_ breath test result reported one or more symptoms: twenty patients reported abdominal pain, seven patients bloating, seven patients diarrhea, and ten patients nausea, heart burn, headache, or dizziness. In total 8 patients (3%) with a positive and 198 patients (75.3%) with a negative H_2_/CH_4_ breath test showed neither gastrointestinal nor extraintestinal symptoms during the breath test.

### 3.6. Lactose-Dependent SIBO (Table 4)

All in all 20 patients (7.6%) with an increase of >20 H_2_ and/or CH_4 _ppm above the baseline during the small intestine transit time (up to 60 minutes after lactose ingestion) were classified as patients with a lactose-dependent SIBO. As shown in Table 4, 16 patients (6.1%) were found with a positive H_2_ breath test and 4 patients (1.5%) with a positive CH_4_ test. No one presented a combined positive H_2_/CH_4_ breath test.

## 4. Discussion

In the present study, the agreement between the H_2_/CH_4_ breath test and the genetic test was only moderate with a Cohen Kappa of 0.44. In contrast previous studies have demonstrated an excellent agreement between the breath test and the genetic test based on the C/T_−13910_ polymorphism [10–12]. Five patients (1.9%) with a negative genetic test were found with a positive H_2_/CH_4_ breath test result. They were categorized as secondary lactose malabsorbers [3, 7], associated with SIBO, gastroenteritis, or colitis ulcerosa. In total 32 patients (12.2%) were found with a positive genetic test and a false negative H_2_/CH_4_ breath test result. Various reasons can lead to false negative breath test results. First of all the H_2_/CH_4_ measurement with the breath test is user-related and depends on preanalytical factors. Poor patient preparation combined with daily changes in the outpatient clinic staff can be a major cause of false negative H_2_/CH_4_ measurements. The end-expiratory alveolar air sample is of importance for the reliability of the breath test. In addition extraintestinal influences such as hyperventilation and exercise can significantly reduce the concentration of the exhaled gases [22, 23]. The previous use of antibiotics may also be a cause of false negative breath tests [24]. Although patients who have completed antibiotic therapy were excluded for at least four weeks from the breath test in this study, alterations of the colonic bacterial flora cannot be completely ruled out. The colonic pH is considered to influence the breath test results. In individuals with carbohydrate malabsorption the colonic contents are often acidic. An acidic colonic microclimate may affect the magnitude of bacterial gas production in the colon and cause false negative breath test results [25, 26]. Another major variable of the breath test is the orocecal transit time. Constitutional and environmental factors affecting the transit time might play an important role in the composition and activities of the colonic flora [27]. A longer orocecal transit time can cause false negative results because the test may be finished before a measurable H_2_/CH_4_ increase is established. If a slow transit time is suspected, additional readings after 150 and 180 minutes should be considered [6].

All in all 95 patients (36.1%) were classified as non-H_2_ producers in the present study. Compared to a previous study of the Medical University Graz, the recognition of non-H_2_ producers was up to 20% of the tested subjects [28]. The H_2_ based breath test is considered to give false negative test results in about 5–15% mainly due to methane production. Therefore a combined H_2_/CH_4_ measurement is expected to improve the diagnosis of malabsorption syndromes and SIBO [29]. The present study results show that only one non-H_2_ producer (0.4%) had a CH_4_ measurement >20 ppm, another four non-H_2_ producers (1.5%) a CH_4_ measurement >10–20 ppm. The high number of non-H_2_ producers (36.1%) compared to the low number of CH_4_ producers (1.9%) is indicative for user-related preanalytical deviations such as handling the gas chromatography measurements or instructing the patients to exhale end-expiratory breath leading to false negative breath test results.

In humans* Methanobrevibacter smithii* is considered to be the major methanogen responsible for measurable CH_4_ during the breath test [30]. All healthy subjects may produce CH_4_ in various concentrations, but only above a threshold-level CH_4_ does appear in the breath [31]. CH_4_ is associated with a slow intestinal transit and constipation [32–35]. A combined H_2_/CH_4_ measurement is considered to improve the diagnosis of lactose intolerance [20, 35], but rarely a CH_4_ cutoff value for a positive CH_4_ breath test is proposed in the literature. According to a previous work [20], a H_2_ and/or a CH_4_ peak >20 ppm over the baseline value was defined as a positive breath test in this study. In another previous study H_2_ and CH_4_ producers were defined as an increase of >12 H_2 _ppm above the baseline and a mean CH_4_ excretion of 2 ppm [35]. A standardized cutoff value to define a H_2_ and/or a CH_4_ producer is still lacking.

In total 57 patients (21.7%) reported one or more symptoms during the H_2_/CH_4_ breath test. Of these abdominal pain was present in 26 patients (45.6%), bloating in 13 patients (22.8%), and diarrhea in 11 patients (19.2%). Lactose intolerance is defined as lactose malabsorption with gastrointestinal symptoms [5, 7]. All in all 16 patients (6.1%) with a positive H_2_/CH_4_ breath test and reported gastrointestinal symptoms were classified as lactose intolerant. Eight patients (3%) with a positive H_2_/CH_4_ breath test result and a lack of clinical symptoms were categorized as lactose malabsorbers. The validity of the reported symptoms during the breath test is limited because the symptom recording and scoring is proposed not only during but also eight hours after the breath test [3, 4, 14]. Furthermore the subjective perception of symptoms that patients associate with lactose intolerance does not always indicate lactose malabsorption [36].

According to the literature [6], 20 patients (7.6%) with a H_2_ and/or CH_4_ peak of >20 ppm above the baseline value during the small intestine transit time (up to 60 minutes after lactose ingestion) were classified as patients with a lactose-dependent SIBO. It is difficult to define the true prevalence of SIBO because of many confounding factors influencing the breath test. The prevalence depends on the nature and the dose of sugar used [16]. Glucose or lactulose H_2_ breath tests are the most commonly used tests [18]. Uniform criteria for breath test interpretation have not been proposed yet [37]. The accuracy and validity of the lactose H_2_/CH_4_ breath test interpretation are limited because this method has not been standardized yet. There is little experience with CH_4_ and it is not clear if the CH_4_ production after sugar ingestion can be interpreted in the same way as the H_2_ production. In the present study four patients (1.5%) with a positive CH_4_ peak within 60 minutes after lactose ingestion were classified as SIBO. All of them had a negative H_2_ breath test; two patients showed intestinal symptoms. Another variable of the breath test is the small intestine transit time. An accelerated or delayed transit time can cause false negative or positive results. The major problem of the diagnosis of SIBO is that no gold standard method has been established yet because the culture of bacteria has its own difficulties and limitations [18, 29].

The limitation of this study is the retrospective study design. Lactose was given in a dose of 50 g. In a recent study 50 g lactose was considered as a nonphysiological dose for the breath test as compared to 25 g [38]. The 50 g lactose dose might have led to a false positive lactose H_2_/CH_4_ breath test result, which was considered as secondary lactose malabsorption. In patients with secondary lactose malabsorption, the lactose breath test was not repeated to verify the recovered enzymatic activity. The symptom recording during the breath test was not carried out with a standardized questionnaire. Additional readings after 150 and 180 minutes were not made. Lactulose or glucose breath tests were not performed to confirm the diagnosis of SIBO. Prospective studies are needed to define uniform criteria for the interpretation of the combined H_2_/CH_4_ breath test and to promote the standardization of this limited diagnostic tool.

## 5. Conclusions

The present results show a moderate agreement between the combined H_2_/CH_4_ lactose breath test and the genetic test (C/T_−13910_ polymorphism) in daily clinical practice. Secondary lactose malabsorption as well as the preanalytical limitations of the breath test procedure and patient preparation can cause discrepant results between both methods. In clinical routine the combined H_2_/CH_4_ breath test as well as the genetic test (C/T_−13910_ polymorphism) should be performed on the one hand to verify patients with a secondary lactose malabsorption and on the other hand to detect false negative breath test results. A standardization of the H_2_/CH_4_ breath test procedure and interpretation is urgently needed to improve the diagnosis of lactose malabsorption and lactose-dependent SIBO.

## Figures and Tables

**Table 1:** Positive lactose H_2_ and/or CH_4_ breath test results.

*n* = 24 (9.1%)	H_2 _breath test	CH_4 _breath test
*n* = 21 (8.0%)	+	−
*n* = 2 (0.7%)	−	+
*n* = 1 (0.4%)	+	+

+: >20 parts per million above baseline during the colon passage (60–120 minutes), –: negative.

**Table 2:** Lactose H_2_/CH_4_ breath test versus genetic test (C/T_−13910  _ polymorphism) results.

	*n* = 263	
	H_2_/CH_4 _breath test^+^	H_2_/CH_4 _breath test^−^
C/C_−13910_	19 (7.2%)	32 (12.2%)
C/T_−13910_	4 (1.5%)	132 (50.19%)
T/T_−13910_	1 (0.4%)	75 (28.51%)

+: >20 parts per million above baseline during the colon passage (60–120 minutes), −: negative.

**Table 3:** Reported symptoms during the lactose H_2_/CH_4_ breath test.

	*n* = 263	
	H_2_/CH_4 _breath test^+^	H_2_/CH_4 _breath test^−^
Symptoms^+^	16 (6.1%)	41 (15.6%)
Symptoms^−^	8 (3%)	198 (75.3%)

+: >20 parts per million above baseline during the colon passage (60–120 minutes), −: negative.

**Table 4:** Lactose-dependent SIBO.

*n* = 20 (7.6%)	H_2 _breath test	CH_4 _breath test
*n* = 16 (6.1%)	+	−
*n* = 4 (1.5%)	−	+

SIBO: small intestinal bacterial overgrowth, +: >20 parts per million above baseline up to 60 minutes after lactose ingestion, −: negative.
